# Proceedings of the 2016 China Cancer Immunotherapy Workshop: Beijing, China. 25-26 June 2016

**DOI:** 10.1186/s13045-016-0322-x

**Published:** 2016-10-13

**Authors:** Bin Xue, Jiaqi Xu, Wenru Song, Zhimin Yang, Ke Liu, Zihai Li, Zihai Li, Lieping Chen, Edward B. Garon, Siwen Hu-Lieskovan, Wei Ding, Chong-Xian Pan, Weijing Sun, Yong-Jun Liu, Lei Zheng, Delong Liu, Michel Sadelain, Cassian Yee, Rongfu Wang, Meixia Chen, Yao Wang, Zhiqiang Wu, Hanren Dai, Can Luo, Yang Liu, Chuan Tong, Yelei Guo, Qingming Yang, Weidong Han, Lisa H. Butterfield, Timothy A. Chan, Wenru Song, Ruirong Yuan, Bo Lu, Ke Liu, Max Ning, Harald Enzmann, Heinz Zwierzina

**Affiliations:** 1 China Center for Food and Drug International Exchange (CCFDIE), Beijing, China; 2 Center for Drug Evaluation (CDE) of the China Food and Drug Administration (CFDA), Beijing, China; 3 Chinese American Hematologist and Oncologist Network (CAHON), New York, NY USA; 4 Department of Microbiology and Immunology, Hollings Cancer Center, Medical University of South Carolina, Charleston, SC USA; 5 Yale University, New Haven, CT USA; 6 David Geffen School of Medicine at UCLA, Los Angeles, CA USA; 7 University of California at Los Angeles, Los Angeles, CA USA; 8 Hematology, Mayo Clinic, Rochester, MN USA; 9 Department of Internal Medicine and Urology, University of California Davis Comprehensive Cancer Center, Sacramento, CA USA; 10 University of Pittsburgh Cancer Center, Pittsburgh, PA USA; 11 Global R&D, Sanofi, Paris, France; 12 Department of Oncology and Surgery, The Sidney Kimmel Comprehensive Cancer Center, Johns Hopkins University School of Medicine, Baltimore, Maryland USA; 13 Skip Viragh Center for Pancreatic Cancer, Johns Hopkins University School of Medicine, Baltimore, Maryland USA; 14 Bloomberg-Kimmel Institute of Cancer Immunotherapy, Johns Hopkins University School of Medicine, Baltimore, Maryland USA; 15 Graduate Program in Cellular and Molecular Medicine, Johns Hopkins University School of Medicine, Baltimore, Maryland USA; 16 New York Medical College and Westchester Medical Center, Valhalla, NY USA; 17 Center for Cell Engineering, Memorial Sloan Kettering Cancer Center, New York, NY USA; 18 Department of Melanoma, MD Anderson Cancer Center, Houston, TX USA; 19 Houston Methodist Research Institute, Houston Methodist Hospital and Weill Cornell Medicine, New Haven, TX USA; 20 Department of Molecule & Immunology/Bio-therapeutic Department, Chinese PLA General Hospital, Beijing, 100853 China; 21 University of Pittsburgh, Pittsburgh, PA USA; 22 Memorial Sloan Kettering Cancer Center, New York, NY USA; 23 Chinese American Hematologist and Oncologist Network (CAHON), New York, NY USA; 24 New Jersey Veteran’s Administration Medical Center, East Orange, NJ USA; 25 Thomas Jefferson University Sidney Kimmel Medical College, Philadelphia, PA USA; 26 Chinese-American Hematologist and Oncologist Network (CAHON), New York, NY USA; 27 Chinese-American Hematologist and Oncologist Network (CAHON), New York, NY USA; 28 Federal Institute for Drugs and Medical Devices, Kurt-Georg-Kiesinger-Allee 3, 53175 Bonn, Germany; 29 Cancer Drug Development Forum - CDDF Headquarters, c/o ECCO - Avenue E. Mounier 83, B-1200 Brussels, Belgium

## Abstract

A1 Proceedings of 2016 China Cancer Immunotherapy Workshop, Beijing, China

Bin Xue, Jiaqi Xu, Wenru Song, Zhimin Yang, Ke Liu, Zihai Li

A2 Set the stage: fundamental immunology in forty minutes

Zihai Li

A3 What have we learnt from the anti-PD-1/PD-L1 therapy of advanced human cancer?

Lieping Chen

A4 Immune checkpoint inhibitors in lung cancer

Edward B. Garon

A5 Mechanisms of response and resistance to checkpoint inhibitors in melanoma

Siwen Hu-Lieskovan

A6 Checkpoint inhibitor immunotherapy in lymphoid malignancies

Wei Ding

A7 Translational research to improve the efficacy of immunotherapy in genitourinary malignancies

Chong-Xian Pan

A8 Immune checkpoint inhibitors in gastrointestinal malignancies

Weijing Sun

A9 What’s next beyond PD-1/PDL1?

Yong-Jun Liu

A10 Cancer vaccines: new insights into the oldest immunotherapy strategy

Lei Zheng

A11 Bispecific antibodies for cancer immunotherapy

Delong Liu

A12 Updates on CAR-T immunotherapy

Michel Sadelain

A13 Adoptive T cell therapy: personalizing cancer treatment

Cassian Yee

A14 Immune targets and neoantigens for cancer immunotherapy

Rongfu Wang

A15 Phase I/IIa trial of chimeric antigen receptor modified T cells against CD133 in patients with advanced and metastatic solid tumors

Meixia Chen, Yao Wang, Zhiqiang Wu, Hanren Dai, Can Luo, Yang Liu, Chuan Tong, Yelei Guo, Qingming Yang, Weidong Han

A16 Cancer immunotherapy biomarkers: progress and issues

Lisa H. Butterfield

A17 Shaping of immunotherapy response by cancer genomes

Timothy A. Chan

A18 Unique development consideration for cancer immunotherapy

Wenru Song

A19 Immunotherapy combination

Ruirong Yuan

A20 Immunotherapy combination with radiotherapy

Bo Lu

A21 Cancer immunotherapy: past, present and future

Ke Liu

A22 Breakthrough therapy designation drug development and approval

Max Ning

A23 Current European regulation of innovative oncology medicines: opportunities for immunotherapy

Harald Enzmann, Heinz Zwierzina

## ᅟ

### A1 Proceedings of 2016 China Cancer Immunotherapy Workshop, Beijing, China

#### Bin Xue^1^, Jiaqi Xu^2^, Wenru Song^3^, Zhimin Yang^2^, Ke Liu^3^ and Zihai Li^3^

##### ^1^China Center for Food and Drug International Exchange (CCFDIE), Beijing, China; ^2^Center for Drug Evaluation (CDE) of the China Food and Drug Administration (CFDA), Beijing, China; ^3^Chinese American Hematologist and Oncologist Network (CAHON), New York, NY, USA

###### **Correspondence:** Bin Xue (xb@ccfdie.org) – China Center for Food and Drug International Exchange (CCFDIE), Beijing, China; Jiaqi Xu (xujq@cde.org.cn) – Center for Drug Evaluation (CDE) of the China Food and Drug Administration (CFDA), Beijing, China; Wenru Song (wenru.song@gmail.com) – Chinese American Hematologist and Oncologist Network (CAHON), New York, NY, USA; Zihai Li (zihai@musc.edu) – Chinese American Hematologist and Oncologist Network (CAHON), New York, NY, USA

The highly anticipated 2016 China Cancer Immunotherapy Workshop held on June 25–26, 2016 in Beijing, China was a huge success. Built on the overwhelmingly positive feedback from the 2015 China Cancer Immunotherapy Workshop, this event continued the tradition of being the leading forum for delivering authoritative and updated knowledge on the rapidly evolving field of immuno-oncology. The workshop represented the ongoing collaboration among three organizations: China Center for Food and Drug International Exchange (CCFDIE), the Center for Drug Evaluation (CDE) of the China Food and Drug Administration (CFDA), and the Chinese American Hematologist and Oncologist Network (CAHON).

Recent years have witnessed rapid and explosive developments in cancer immunotherapy which has been viewed as a “historic breakthrough” in cancer medicine. Scientists and physicians are engaged at the incredible pace in discovery and clinical development of new cancer immunotherapeutic agents. Patients eagerly expect more options for immunotherapy and they anticipate significant clinical benefit, even cure. In the meantime, these new advances and challenges necessitate physicians, scientists, and regulators to work together in an unprecedented way to learn and keep abreast of the latest developments in the field.

World-leading experts from academia, regulatory agencies and industries shared their views and experiences in several thematic areas, organized into the following seven sessions: (1) basic immunology and cancer immunology; (2) clinical updates on checkpoint inhibitors; (3) emerging new immunotherapy; (4) perspective from industry; (5) clinical updates on cellular therapy; (6) unique clinical development considerations; and (7) regulatory considerations. As a result of demand from conference participants, the conference organizers decided to publish the proceedings of the meeting to benefit a broader readership of the *Journal of Hematology and Oncology* on the topic. Abstracts of all presentations are included except talks in Session 4 which highlighted the immune-oncology pipelines of the following biotechnology and pharmaceutical companies: Bristol-Myers Squibb, Merck, Roche/Genetech, AstraZeneca, Merck Sereno, Pfizer, Amgen, BeiGene, Henri, JW Biotechnology and InnoVent. The information is publically available in their respective websites.

## Session 1: Basic Immunology and Cancer Immunology

### A2 Set the stage: fundamental immunology in forty minutes

#### Zihai Li (zihai@musc.edu)

##### Department of Microbiology and Immunology, Hollings Cancer Center, Medical University of South Carolina, Charleston, SC, USA

The immune system in mammals is a hard-wired defense mechanism that has co-evolved with the host to defend against pathogens, histoincompatible antigens and altered ‘self’ as a result of malignant transformations and other damages. Functionally, the immune system is often considered to have the innate and the adaptive arms. Composed of physical barriers such as the skin and gut mucus layer, as well as lytic enzymes, cytokines, and phagocytic cells such as macrophages and dendritic cells, innate immunity is a built-in system ready for immediate actions against pathological insults without the need for pre-sensitization. The receptors to activate the innate immunity such as Toll-like receptors, Nod-like receptors and cytosolic DNA sensors are germline-encoded gene products that can recognize a broad range of shared molecular moieties in the pathogens or damaged tissues. T and B lymphocytes are key cell types in the adaptive immunity that have exquisite mono-specificity (clonality) against antigens due to unique clonal receptors on their surface generated from gene recombination at the somatic level. The formation of antigen receptors by gene recombination ensures the development of a diverse repertoire of T and B cells for recognizing practically all possible antigens. Depending on expression of the co-receptor molecules CD4 and CD8, T cells can be divided into CD4+ and CD8+ cells which recognize peptide antigens in association with MHC class II and class I molecules respectively. A key function of CD4+ cells is to help CD8+ cells to attain effector functions (such as cytotoxicity) and assist B cells to differentiate into plasma cells and make immunoglobulins (including five Ig isotypes: IgG, IgA, IgD, IgM and IgE) that recognize antigens directly without the need for MHC molecules. One hallmark of T and B cells is that they can be programed into memory cells, which mount a greater response upon secondary exposure to the same antigens. Importantly, innate and adaptive immunity do not operate in isolation. Recent exciting progress in fundamental immunology has uncovered the following guiding principles of immunology that are particularly relevant for clinical translation in oncology: (1) The activation of innate immunity, particularly dendritic cells, is often the prerequisite for initiation of adaptive immunity, which is the reason why adjuvant is needed for optimal vaccinations. (2) Depending on the quality of the innate signals, adaptive immunity can adopt a distinct functional fate, favoring for instance helper T cell response (Th) of different types (Th1, Th2, etc.). (3) Adaptive immunity must always be kept in check by both central and peripheral tolerance mechanisms such as negative checkpoint signals and regulatory T cells due to the inherent risk of the adaptive immunity to inflict autoimmunity. (4) Cancers or chronic infectious agents take advantage of tolerance mechanisms to evade the host immune defense. In short, it is ripe to harness the immune system for combating human diseases due to increasing understanding of the mechanisms that govern several laws of the system: diversity, tolerance, memory and appropriateness.

### A3 What have we learnt from the anti-PD-1/PD-L1 therapy of advanced human cancer?

#### Lieping Chen (lieping.chen@yale.edu)

##### Yale University, New Haven, CT, USA

Immune responses are tightly controlled by cell surface immune modulatory molecules that constitute various receptors and ligands and could positively or negatively influence the quality and even the direction of immune responses. The PD-1/PD-L1 immune modulatory pathway plays important roles in suppressing antigen-specific immune responses and inflammation. Selective expression of PD-L1 (B7-H1) in tumor microenvironment and subsequent interaction with PD-1 on tumor-infiltrating T cells is demonstrated to be a major mechanism of losing T cell immunity in tumor sites in a significant fraction of cancer patients, a mechanism called adaptive resistance. Monoclonal antibodies blocking this pathway have been tested broadly worldwide and induced regression of a broad spectrum of advanced human cancers especially solid tumors. The treatment is well-tolerated and the clinical responses could be long-lasting. In the context of ongoing large-scale clinical trials in thousands of cancer patients, understanding of immunological mechanisms underlying response and resistance to anti-PD-1/PD-L1 therapy is critical for further improvement of cancer therapy in the future. I will discuss principles of anti-PD-1/PD-L1 therapy and perspectives on cancer therapy using this immune modulation approach.

*Disclosure: Scientific advisory board-Pfizer, AstraZeneca, Johnson & Johnson, NextCure; GenomiCare; Research funding- Boehringer Ingelheim, Pfizer, NextCure.*

## Session 2: Clinical Updates on Checkpoint Inhibitors

### A4 Immune checkpoint inhibitors in lung cancer

#### Edward B. Garon (egaron@mednet.ucla.edu)

##### David Geffen School of Medicine at UCLA, Los Angeles, CA, USA

Immune checkpoint inhibitors have rapidly become an established therapy for patients with non-small cell lung cancer (NSCLC). In the United States, two inhibitors of the programmed cell death 1 (PD-1) immune checkpoint, nivolumab and pembrolizumab, are approved for the treatment of previously treated NSCLC. Each drug has an approved diagnostic test for the PD-1 ligand PD-L1. The role of PD-L1 testing remains controversial, not necessarily based on doubts as to whether high PD-L1 levels enhance the likelihood of benefit from PD-1 inhibitors in non-squamous NSCLC, but rather whether patients with low or absent staining should still get PD-1 inhibitors in light of available alternate approaches in previously treated NSCLC. Although potential differences in the PD-L1 tests have been a concern to date, recent studies have shown strong similarity for the available tests in NSCLC specimens. Data is also evaluating a host of alternate and/or complementary biomarkers in addition to PD-L1. Emerging data is assessing the role of several PD-L1 inhibitors in NSCLC. In addition, emerging data is evaluating inhibitors of the PD-1 checkpoint in a variety of NSCLC clinical settings, including frontline therapy for metastatic disease, adjuvant therapy, and consolidation therapy after chemoradiotherapy for locally advanced disease. There is also emerging data in small cell lung cancer, although the number of patients evaluated to date is relatively small. The role of inhibitors of other immune checkpoints in lung cancer are less certain to date. Many clinical trials evaluating alternate checkpoint inhibitors or combinations of inhibitors of the PD-1 checkpoint with inhibitors of other checkpoint are underway. The combination of inhibitors of the PD-1 checkpoint and cytotoxic T-lymphocyte associated protein 4 (CTLA-4) have been the most extensively evaluated combination to date, with data that is promising, but limited by additional toxicity, small numbers of patients in non-randomized studies and inconsistent results with respect to the role of PD-L1 in predicting which patients will benefit.

### A5 Mechanisms of response and resistance to checkpoint inhibitors in melanoma

#### Siwen Hu-Lieskovan (shu-lieskovan@mednet.ucla.edu)

##### University of California at Los Angeles, Los Angeles, CA, USA

Recent breakthrough in immunotherapy for cancer provides potential of long lasting benefit to patients with melanoma and a wide array of other tumor subtypes. Inhibiting adaptive immune resistance is the mechanistic basis of the antitumor activity of PD-1 immune checkpoints blockade therapies, by releasing the breaks or unleashing the immune system in patients whose immune system was ready to attack the cancer but was being blocked by the cancer. Combination therapies to increase the priming of T cells and overcome the immune-suppressive tumor microenvironment are being developed to increase the benefit to more tumor types and patients. Engineering the immune system for adoptive cell transfer (ACT) therapy is being developed to help patients who cannot mount a tumor specific immune response. Genetic (and likely epigenetic) alterations leading to crippled IFN-receptor signaling result in primary resistance to PD-1 blockade therapy. Loss of function mutations in IFN-receptor signaling or antigen presenting machinery mediate acquired resistance to PD-1 blockade therapy.

### A6 Checkpoint inhibitor immunotherapy in lymphoid malignancies

#### Wei Ding (ding.wei@mayo.edu)

##### Hematology, Mayo Clinic. Rochester, MN, USA

The inhibitors of checkpoint pathways aiming to unleash the brake on immune system have been tested in multiple solid tumors and have revolutionized cancer therapy in the last five-ten years. However, the clinical trials of testing checkpoint inhibitors in hematological malignancies have just been initiated and are still in their infancies. Recent early phase trials of testing PD-1 blocking antibody nivolumab or pembrolizumab in relapsed and refractory hodgkin’s lymphoma have revealed robust clinical response with an estimated overall response rate 65-85 %. However, the single- agent response rate of PD-1 blockade in multiple subtypes of non-Hodgkin’s lymphoma (NHL) was only 10-40 %. The etiology of these differential responses has just been partially elucidated. Genetic alterations causing amplification or translocation of chromosome 9p24.1 where PD-L1 and PD-L2 genes are located have been detected in classical hodgkin’s lymphoma and primary mediastinal large B cell lymphoma. Expression of PD-L1 are also present in multiple types of other NHL including T cell rich large cell lymphoma, post-transplant lymphoproliferative disorder, EBV positive lymphoma including diffuse large B cell lymphoma. Studies focusing on dissecting tumor microenvironment in lymph nodes have revealed complex interactions and differential bias of T cell immunity in different subtypes of lymphoma. Biomarkers that can predict clinical response of PD-1 blockade are needed in many types of lymphomas. Further combination therapies of signal inhibitors with immunotherapy are intensely studied in the ear of novel immunotherapy to improve clinical efficacies.

### A7 Translational research to improve the efficacy of immunotherapy in genitourinary malignancies

#### Chong-Xian Pan (cxpan@ucdavis.edu)

##### Department of Internal Medicine and Urology, University of California Davis Comprehensive Cancer Center, Sacramento, CA, USA

Immunotherapy with checkpoint inhibitors has shown promising activity in genitourinary malignancies. In kidney cancer, the anti-Programmed Death 1 (PD1) antibody nivolumab has been approved by the US Food and Drug Administration (FDA) to treat patients with metastatic renal cell carcinoma after progression on an antiangiogenic therapy. The anti-tumor activity can be seen in both PD1-positive and PD1-negative kidney cancers. In prostate cancer, even though Sipuleucel-T is approved for the treatment of castration-resistant metastatic prostate cancer with minimal or no symptoms, its treatment is not associated with any improvement of clinical or objective progression-free survival or prostate-specific antigen (PSA) reduction. In urothelial cancer, an anti-programmed death ligand 1 (PD-L1) antibody atezolizumab has been approved for the treatment of patients with locally advanced or metastatic urothelial carcinoma whose disease has worsened during or following platinum-containing chemotherapy, or within 12 months of receiving platinum-containing chemotherapy, either before (neoadjuvant) or after (adjuvant) surgical treatment. However, only a minority of bladder cancer patients benefit atezolizumab. PD-L1 expression, luminal subtype II and high mutation load are associated with higher response rate to atezolizumab. Nevertheless, cancer response is also observed in cancer with absence of PD-L1 expression, other urothelial cancer subtypes and low mutation load. In addition to the above mentioned agents, several other checkpoint inhibitors have also showed anti-tumor activity. Many clinical trials are currently going to determine whether the efficacy of immunotherapy can be improved by combining a checkpoint inhibitor with other immunoregulatory agents, chemotherapy, radiation therapy or targeted therapy.

Despite the above mentioned great achievement, lack of appropriate animal models hinders research in immunotherapy. To address this issue, University of California Davis and The Jackson Laboratory have collaborated to establish humanized mice carrying patient-derived xenografts (PDXs). Humanized mice were generated and human immune system was reconstituted after injection of human hematopoietic cells into immunodeficient NSG mice. PDXs were developed after implantation of uncultured tumor specimens from human patients into NSG mice. PDXs retained the morphology fidelity and 92-97 % genetic alterations of parental patient cancers. Humanized mice carrying urothelial cancer PDXs responded to an anti-PD1 antibody pembrolizumab similar to that observed in clinical patients. Treatment of pembrolizumab was associated with infiltration of CD45 + CD8+ human T cells in PDXs. Other molecular correlative studies are currently going on. In order to improve the treatment of urothelial cancer, we developed urothelial cancer-specific porphyrin-based nanoparticles. These cancer-specific nanoporphyrin can be used for targeted chemotherapy, radiation therapy, photodynamic therapy and photothermal therapy. Research is currently being performed to determine whether these targeted therapies with cancer-specific nanoporphyrin can improve the efficacy of immunotherapy.

### A8 Immune checkpoint inhibitors in gastrointestinal malignancies

#### Weijing Sun (sunw@upmc.edu)

##### University of Pittsburgh Cancer Center, Pittsburgh, PA, USA

Gastrointestinal (GI) malignancies are highly aggressive and heterogeneous diseases. The overall outcomes of advanced and metastatic GI malignancies are still very disappointing with the current chemotherapy options. Target-oriented agents have only showed moderate effectiveness. Although the attempt of immunotherapy has been for many years, it is not only until very recent years that the clinically encouraging results begun to emerge, primarily in the areas of immune checkpoints blockade against PD-1/PD-L1 and CTLA4 in management of GI cancers.

Microsatellite instability (MSI) plays a significant role in the GI cancers’ formation and development, and is characterized by deletion or mutations of DNA mismatch repair (MMR) genes (e.g., Mlh1, Msh2, Pms1 and Pms2), or the hypermethylation of the promoters of the genes (e.g., Mlh1). The high mutational load in MSI (MMR-deficiency) tumors are therefore associated with high level of tumor-specific neoantigens, which are frequently recognized by the immune system. A phase II study showed a good activity of pembrolizumab, an anti-PD-1 monoclonal antibody in patients with chemotherapy-refractory metastatic GI malignancies with MSI. Furthermore, a recent reported interim data analysis of the study (Checkmate 142) showed the advantage of combining the anti-PD-1 monoclonal antibody (Nivolumab) with anti-CTLA monoclonal antibody (Ipilimumab) in MMR-deficient colorectal cancer over anti-PD-1 alone. The efficacy of anti-PD-L1 in combination with MEK inhibitor showed very encouraging results in Kras muted metastatic colorectal cancer.

Based on the classification by the Cancer Genome Atlas (TCGA) project, four major molecular subtypes are recognized in gastric cancer: chromosomally unstable, Epstein Barr Virus (EBV)-infection related, MSI-associated, and genomically stable disease. In the EBV-infection related subgroup, PD-L1 and PD-L2 are upregulated due to chromosome 9p24 amplification. Therefore, blockading the PD-1 or PD-L1/PD-L2 in this subgroup is likely to create recognizable antitumor activity. Efficacy of anti-PD-I monoclonal antibodies (nivolumab or pembrolizumab) has been demonstrated in patients with advanced/metastatic esophageal and gastric cancer with or without PD-L1 expression (Keynote-012, Keynote-028 and CheckMate-032). More studies are ongoing at phase II and III levels in different settings.

Hepatocellular microenvironment is characterized by immunosuppression with PD-L1 expression on Kupffer cells and on sinusoidal endothelial cells. Preliminary phase I study data suggested very encouraging results of nivolumab in the treatment of patients with advanced hepatocellular carcinoma (HCC) with various etiology, including those caused by alcohol and viruses (both hepatitis B and C viruses).

In summary, immune therapy has shown encouraging results in the treatment of GI malignances and likely has a bright future. Current evidences are derived mainly from the immune checkpoint blockade monotherapy. Future studies are warranted in testing immune checkpoint blockers in combination with chemotherapy, targeted agents, as well as other immunotherapeutic strategies.

## Session 3: Emerging New Immunotherapy

### A9 What’s next beyond PD-1/PDL1?

#### Yong-Jun Liu (yong-jun.liu@sanofi.com)

##### Head of Research, Global R&D, Sanofi, Paris, France

The current excitement of immuno-oncology stems from decades of basic research in immunology. There are five essential steps to elicit effective anti-cancer T cell immunity: (1) tumor antigen presentation by dendritic cells (DCs); (2) priming of naïve T cells by immunogenic DCs; (3) egress of effector T cells from the secondary lymphoid organs; (4) infiltration of tumors by tumor-specific T cells; and (5) eradication of tumors by effector mechanism of T cells. Thus rational strategies are being developed for cancer immunotherapy to boost each of the five processes. I will discuss especially two areas of research in my laboratory. The first has to do with our discovery of plasmacytoid dendritic cells (pDCs) which is a key cell type in conferring anti-viral immunity and therefore plays critical roles in immune surveillance against viral associated malignancies. The second line of investigation deals with regulatory T cells via blocking OX40 receptor. We have generated a monoclonal antibody against OX40. This antibody has been shown to inhibit the immunosuppressive function of Interleukin 10 (IL-10) producing CD4+ type 1 regulatory T cells (“Tr1 cells”) and Foxp3 + −expressing regulatory T cells. Clinical trials are ongoing with this agent. Thus, it is abundantly clear that the field of immuno-oncology will continue to move forward rapidly beyond PD-1/PD-L1 checkpoints.

### A10 Cancer vaccines: new insights into the oldest immunotherapy strategy

#### Lei Zheng (lzheng6@jhmi.edu)

##### ^1^Department of Oncology and Surgery, The Sidney Kimmel Comprehensive Cancer Center, Johns Hopkins University School of Medicine, Baltimore, Maryland; ^2^Skip Viragh Center for Pancreatic Cancer, Johns Hopkins University School of Medicine, Baltimore, Maryland; ^3^Bloomberg-Kimmel Institute of Cancer Immunotherapy, Johns Hopkins University School of Medicine, Baltimore, Maryland; ^4^Graduate Program in Cellular and Molecular Medicine, Johns Hopkins University School of Medicine, Baltimore, Maryland

Immunotherapy is considered to be one of the breakthroughs for cancer treatments in the last decade, attributed to the development of immune checkpoint inhibitors. The first immunotherapy was conducted by Dr. William Coley in 1891 by using a cancer vaccine-like strategy. Nevertheless, the recent results of the clinical trials with cancer vaccine approaches are disappointing. Multiple layers of immune tolerance mechanisms have made the cancer vaccine strategy fail to induce adequate anti-tumor response. On another hand, more than half of the cancer patients do not respond to the single agent immune checkpoint inhibitors due to lack of effector immune cells infiltrating the tumors. Cancer vaccine treatments may prime the immune quiescent tumors with the infiltration of effector immune cells, which also induce the immune checkpoint signals including PD-1/PD-L1 presumably through adaptive resistance mechanisms. The combination of vaccine and immune checkpoint inhibitors may overcome the resistance to the immune checkpoint inhibitors as a single agent treatment. Immune checkpoint inhibitors achieve the anti-tumor activity by unleashing response to tumor neoantigens, suggesting that cancer vaccines that target neoantigens could be more potent. Neoantigens-based cancer vaccines are being developed. In addition, oncolytic virus has been shown to induce immunogenic tumor lysis and can also be employed as a vaccine strategy to induce the immune response to cancer neoantigens. Finally, the combination immunotherapy is the key to the success in making more and more patients benefit from the immunotherapy.

*Disclosure: GVAX – Under a licensing agreement between Aduro BioTech, Inc. and the Johns Hopkins University, the University and investigators (L.Z.) are entitled to milestone payments and royalty on sales of the vaccine product. L.Z. served in the advisory board for Halozyme: advisory board, and received the research grant from Halozyme, BMS, Merck, and iTeos. L.Z. is a paid consultant at Percans and Lifemax.*

### A11 Bispecific antibodies for cancer immunotherapy

#### Delong Liu (delong_liu@nymc.edu)

##### New York Medical College and Westchester Medical Center, Valhalla, NY, USA

Immune checkpoint blockers and CAR-T immunotherapeutic modalities are revolutionizing cancer treatment. Bispecific antibodies offer additional treatment options as cancer immunotherapy [1]. Bispecific T cell engager (BiTE) antibodies are entering clinical applications for pre-B acute lymphoid leukemia (blinatumomab) and certain EpCAM+ solid tumors (catumaxomab) [2–4]. More and more bispecific antibodies are in active clinical development. AFM11, a tetravalent BiTE antibody against CD19 and CD3, is being explored for B cell malignancies. AFM13 with bispecificity toward CD30 and CD16A is pushing NK cells into the field of cancer immunotherapy. AFM13 is tetravalent, and has a longer half life than bivalent BiTE antibodies [5]. Specifically, 28 patients with highly refractory Hodgkin lymphoma have been treated in a phase I trial [6]. Dose limiting toxicity with hemolytic anemia was reported in one patient. Newer technology in antibody design and production make it possible for rapid development of novel antibodies for clinical application.

**References**

1. Fan G, Wang Z, Hao M, Li J: **Bispecific antibodies and their applications**. *Journal of Hematology & Oncology* 2015, **8**(1):130.

2. Wu J, Fu J, Zhang M, Liu D: **Blinatumomab: a bispecific T cell engager (BiTE) antibody against CD19/CD3 for refractory acute lymphoid leukemia**. *Journal of Hematology & Oncology* 2015, **8**(1):104.

3. Linke R, Klein A, Seimetz D: **Catumaxomab: clinical development and future directions**. *MAbs* 2010, **2**(2):129–136.

4. Topp MS, Gokbuget N, Stein AS, Zugmaier G, O'Brien S, Bargou RC, Dombret H, Fielding AK, Heffner L, Larson RA, Neumann S, Foa R, Litzow M, Ribera JM, Rambaldi A, Schiller G, Bruggemann M, Horst HA, Holland C, Jia C, Maniar T, Huber B, Nagorsen D, Forman SJ, Kantarjian HM: **Safety and activity of blinatumomab for adult patients with relapsed or refractory B-precursor acute lymphoblastic leukaemia: a multicentre, single-arm, phase 2 study**. *Lancet Oncol* 2014, **16**(1):57–66.

5. Wu J, Fu J, Zhang M, Liu D: **AFM13: a first-in-class tetravalent bispecific anti-CD30/CD16A antibody for NK cell-mediated immunotherapy**. *Journal of Hematology & Oncology* 2015, **8**(1):96.

6. Rothe A, Sasse S, Topp MS, Eichenauer DA, Hummel H, Reiners KS, Dietlein M, Kuhnert G, Kessler J, Buerkle C, Ravic M, Knackmuss S, Marschner J-P, Pogge von Strandmann E, Borchmann P, Engert A: **A phase 1 study of the bispecific anti-CD30/CD16A antibody construct AFM13 in patients with relapsed or refractory Hodgkin lymphoma**. *Blood* 2015, **125**(26):4024–4031.

## Session 5: Clinical Update on Cellular Therapy

### A12 Updates on CAR-T immunotherapy

#### Michel Sadelain (m-sadelain@ski.mskcc.org)

##### Center for Cell Engineering, Memorial Sloan Kettering Cancer Center, New York, NY, USA

T cell engineering provides a means to rapidly generate therapeutic T cells of any specificity. This novel therapeutic modality is predicated on the transduction of receptors to redirect T cell specificity and enhance T cell function. Chimeric antigen receptors (CARs) are synthetic receptors that mediate antigen recognition, T cell activation, and, in the case of second generation CARs, costimulation. We demonstrated over a decade ago that human T cells engineered with a CD19-specific CAR eradicated B cell malignancies in mice, and we were the first to report remarkable complete remission rates obtained with second generation CD19 CARs in adults with chemorefractory, relapsed acute lymphoblastic leukemia. We have by now infused over 50 patients at our center, who were treated with a single infusion of autologous T cells expressing the 19-28z CAR. The complete response rate is in the 80-90 % range, with over 80 % achieving a molecular response. Within the latter, the estimated 6-month overall survival rate for the minimal (<5 % blasts) and morphologic disease patients (≥5 % blasts) were 92 % and 65 %, respectively, with remissions extending beyond 3 years in the minimal disease cohort. Several groups, including ours, have extended these results to other B cell malignancies including non-Hodgkin lymphoma, pediatric ALL and chronic lymphocytic leukemia. CAR toxicities include B cell aplasia, cytokine release syndrome (CRS) and neurotoxicity. Novel T cell engineering modalities, including auto- and trans-costimulation and combinatorial antigen recognition, hold the promise of further enhancing the effectiveness and safety of CAR therapy against a broad range of cancers. The success of CD19 CAR therapy has generated unprecedented enthusiasm for cell-based therapies, which urgently require the development of more performing and safe manufacturing processes to meet the anticipated demand for autologous and alternative T cell products.

### A13 Adoptive T cell therapy: personalizing cancer treatment

#### Cassian Yee (cyee@mdanderson.org)

##### Department of Melanoma, MD Anderson Cancer Center, Houston, TX, USA

Adoptive cellular therapy (ACT) represents an increasingly attractive treatment for patients with cancer because of the potential for significant anti-tumor efficacy, minimal toxicity and longterm immunoprotection. We have been exploring one form of ACT, known as endogenous T cell (ETC.) therapy Endogenous T cell (ETC.) therapy exploits enabling technology developed in our lab to isolate rare (<1:100,000) antigen specific T cells from the peripheral blood and render them, by IL-21 exposure during priming, to become helper-independent, antigen-driven autocrine central memory type T cells with high replicative capacity and longterm in vivo persistence.

To overcome potential barriers to effective ACT, a means to enhance the duration and efficacy of transferred T cells, modulate and/or eradicate Tregs and lower the threshold of activation of endogenous tumor-reactive effectors would be desirable. We report here the use of a first-in-human adoptive cellular therapy regimen combining the use of IL-21-primed antigen-specific T cells, with a post-infusion course of anti-CTLA4 therapy that can yield long-lasting, and significant clinical responses with minimal toxicity (Chapuis & Yee *et al.,* JEM 2016 and JCO 2016). One major challenge however to advancing the use of ACT for solid tumor malignancies in general is a lack of knowledge of the MHC-restricted peptides presented on individual patient tumors that can be targeted with T cells, and, a means of rapidly deploying antigen-specific adoptive cellular therapy strategy following identification of such immunogenic peptides. Our research group has been focused on developing antigen-specific T-cell based immunotherapies for the treatment of cancer patients [1].

First, by employing a combination of next generation sequencing, mass spectrometry-based proteomics, and HLA bioinformatics, we have developed a highly sensitive tumor antigen identification method that can reliably identify HLA-bound peptide ligands presented by cancer cells from individual patients [2]. Second, we have developed enabling technologies that allow us to successfully isolate and enrich from the endogenous T cell population in patient peripheral blood - rare, tumor antigen-specific T cells recognizing these potential immunogenic epitopes. In preliminary studies, we have routinely generated CTLs specific for tumor antigens identified through our antigen discovery pipeline, and demonstrated anti-tumor activity against HLA-matched tumor cell lines. In contrast to TIL therapy or CAR/TCR-engineered T cells, ETC. therapy provides the expediency and flexibility required to generate T cells against individualized neo-epitopes identified ad hoc from patient tumor samples. We propose to develop personalized, antigen-specific adoptive T cell therapy for patients using this immunopeptidome pipeline and tetramer-guided cell sorting for patients with solid tumor malignancies using a combined ACT + ICI outpatient regimen that has already demonstrated in early studies to be safe, effective, and offers longterm protection from relapse.

**Fig. 1 (abstract A13).**
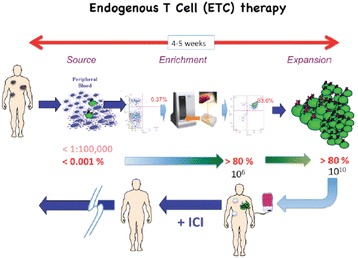
Endogenous T Cell (ETC) therapy

### A14 Immune targets and neoantigens for cancer immunotherapy

#### Rongfu Wang (rwang3@tmhs.org)

##### Houston Methodist Research Institute, Houston Methodist Hospital and Weill Cornell Medicine, New Haven, TX, USA

Harnessing the immune system to eradicate malignant cells is becoming a powerful new approach to cancer therapy. Immunotherapy-based drugs, including anti-CTLA-4, anti-PD-1 and anti-DP-L1antibodies, have been approved for the treatment of many types of cancer. Furthermore, recent clinical trials using antigen-specific T cell receptor (TCR), or CD19-chimeric antigen receptor (CAR), have shown promising clinical results for patients with metastatic cancer. Identification and clinical evaluation of new checkpoint signaling molecules are the major focus for developing new therapeutic drugs. Because these immunotherapies rely on tumor-specific T cells to fight against cancer, recruitment and trafficking of tumor reactive T cells to tumor sites and their persistence have become the one of the most active and fast-moving research areas. For TCR and CAR T cell therapy, the choice of cancer antigens recognized by T cells is pivotal to success of cancer immunotherapy. Despite significant progress in cancer antigen discovery, there are only very few targets that have been demonstrated to mediate clinical response and tumor regression without evident toxicity. NY-ESO-1 is one of such cancer antigens that show the promising clinical activity. NY-ESO-1-specific TCR-engineered T cells have produced 55-80 % of clinical response in metastatic sarcoma, melanoma and myeloma. A key question is how to identify many more immune targets that are suitable for cancer immunotherapy using TCR or CAR technologies for different types of cancer. With recent technology advances in next-generation sequencing, it become possible to dissect the immune response to patient-specific mutated antigens, which may be critical in mediating tumor-specific immune response against cancer. Understanding of these mutated and nonmutated antigens is critical for the development of novel personalized immunotherapy and precision medicine. I will present the latest findings and discuss the current progression and future directions in cancer immunotherapy.

### A15 Phase I/IIa trial of chimeric antigen receptor modified T cells against CD133 in patients with advanced and metastatic solid tumors

#### Meixia Chen, Yao Wang, Zhiqiang Wu, Hanren Dai, Can Luo, Yang Liu, Chuan Tong, Yelei Guo, Qingming Yang, Weidong Han

##### Department of Molecule & Immunology/Bio-therapeutic Department, Chinese PLA General Hospital, Beijing, 100853, China

###### **Correspondence:** Weidong Han (hanwdrsw@sina.com) – Department of Molecule & Immunology/Bio-therapeutic Department, Chinese PLA General Hospital, Beijing, 100853, China

CD133 is well-documented to be expressed by tumor initiating cells and epithelial progenitor cells, which were proposed to have predominant roles for tumor recurrence and pre-metastatic niche formation, respectively. Thus, targeting CD133 might help eradicate the primary tumors and even prevent tumor metastasis. Herein, CD133-directed chimeric antigen receptor modified T cells (CART-133) were successfully generated and their marked antitumor activity was verified. Results from hematopoietic colony forming assays suggested that CART-133 cells may pose no irreversible myelosuppression. From October of 2015 to February of 2016, 10 patients with advanced and sorafenib-refractory hepatocellular carcinoma (HCC) were enrolled on phase I trial and were assigned into 3 dose-escalated cohorts (according to CART-133 positive cell amount: 0.10-1.0 × 106/kg, 2-5 × 106/kg, and 0.5-1.0 × 107/kg). 8 out of 10 patients received CART-133 monotherapy once or repeated infusion every 4–8 weeks. Patients who received CART-133 infusion were assessed for response. All patients had tolerable febrile syndromes during cell infusions. Of consecutively enrolled patients, rapid ascites growth occurred in 1 patient during infusion and was reversible by the use of diuretic, and 1 patient developed transiently drastic decline of hemoglobin and platelets and Grade 3 direct hyperbilirubinemia within 2 weeks after cell infusion. Reverse correlation between CD133+ cells in peripheral blood and CAR copy number in cohort 2 and 3 revealed an effective biological activity and safety of CART-133 and its rational expansion dose. 1 of 3 cases in cohort 1 aggressively progressed after cell therapy and became stable after transferred to cohort 2. Seven cases maintained stable disease as of the most recent follow-up, however, 2 patients died of upper gastrointestinal massive hemorrhage >9 weeks after infusion. On the basis of the presented data, additional 13 patients (5 sorafenib refractory HCCs, 5 advanced/metastatic pancreatic carcinomas, 2 metastatic colorectal carcinomas, and 1 advanced cholangiocarcinoma) were recruited into phase II trial using the expansion dose so far. All toxicities associated with the cell therapy even in those who received chemo-combined regimens with multiple cycle CART-133 infusions were controllable. The clinical response evaluation of all these patients in phase 2 is still ongoing. This study demonstrated the safety, feasibility, and preliminarily clinical efficacy of CART-133 treatment in epithelium-derived solid tumors, and guaranteed further patient recruitment. This clinical trial is registered at www.clinicaltrials.gov as NCT02541370.

*All authors declare that they have no competing interests. Correspondence to: Weidong Han, MD, PhD, Department of Molecule & Immunology/Bio-therapeutic Department, Chinese PLA General Hospital, Beijing, 100853, China. E-Mail: hanwdrsw@sina.com. or hanwdrsw69@yahoo.com*

## Session 6: Unique Clinical Development Considerations

### A16 Cancer immunotherapy biomarkers: progress and issues

#### Lisa H. Butterfield (butterfieldl@upmc.edu)

##### University of Pittsburgh, Pittsburgh, PA, USA

Biomarkers that can 1) predict which patient should be enrolled in a clinical trial or receive a treatment; 2) prognosticate early on treatment whether a patient is receiving benefit from therapy; or 3) identify the mechanism of action of a therapeutic intervention are critically needed in immunotherapy for cancer. Because immunotherapy involves manipulation of a complex network of cells and molecules throughout the body (and not changing the function of a single protein), identification of these biomarkers is difficult. Despite many years of use of interferon and IL-2 in melanoma and other cancers, the patients who can and will benefit and the exact mechanism of action is still not known. Importantly, research lab-based and retrospective biomarker studies are further complicated by lack of standardization of patient blood and tumor sample processing and storage. There are many candidate biomarker assessments that have given useful and important data regarding patient outcomes, including testing tumor-specific T cell frequencies and T cell activation, and suppressive cell measures (including regulatory T cells (Treg) and myeloid-derived suppressor cells (MDSC)), in blood and in tumors. In some cases, phenotypic measures without functional testing can give conflicting results. Newer molecularly-based assays of tumor mutation load, T Cell Receptor (TCR) diversity and tumor inhibitory molecule expression (like PD-L1 staining) are giving interesting signals that require further investigation in multiple tumor settings and at multiple time points to further substantiate. The Society for Immunotherapy of Cancer (SITC) has lead several initiatives over the last 15 years in immunologic monitoring in cancer. Most recently, the Immunoscore task force has completed their work, and the Immunologic Biomarkers Task Force have prepared white papers on the state of the art, hurdles in the field and recommendations for the future that are now being published to help the field progress in the identification of these important biomarkers.

*Disclosures: Stock options: Kite Pharma; Advisory Board participation: Oxford Immunotec, Affymetrix/eBioscience, Merck, Biodesix, Verastem, AstraZeneca*

### A17 Shaping of immunotherapy response by cancer genomes

#### Timothy A. Chan (chant@mskcc.org)

##### Memorial Sloan Kettering Cancer Center, New York, NY, USA

Immune checkpoint blockade is a promising approach for the treatment of human malignancies. For example, treatment of patients with advanced lung cancers and melanoma have resulted in improved response rates and durable disease control. However, the extent to which patients derive benefit is diverse and the determinants that drive response to therapy are ill-defined. We have sought to define the genomic determinants of response to immune checkpoint blockade therapies such as anti-CTLA-4 and anti-PD-1. Our work has shown that tumor mutational burden, clonality, and mutational landscape features help dictate clinical response. Mutations in genes that are part of the antigen presentation machinery are rare but can be preferentially downregulated in tumors. Reexpression of genes in the MHC antigen presentation pathway by treatment with epigenetic therapy synergizes with immune checkpoint blockade to boost anti-tumor responses.

### A18 Unique development consideration for cancer immunotherapy

#### Wenru Song (wenru.song@gmail.com)

##### Chinese American Hematologist and Oncologist Network (CAHON), New York, NY, USA

Cancer immunotherapy has progressed very rapidly in the past few years, with the potential to transform future new standard of care in oncology due to its unique science and its potential for substantial and long-term clinical benefit. The success is based on the progress in both preclinical and clinical science, including the development of new paradigm of clinical investigation. As the target of immunotherapy is not directly attacking the tumor but instead mobilizing the host immune system, the unique development consideration (study endpoint, safety, trial design, etc.) of cancer immunotherapy will be discussed.

### A19 Immunotherapy combination

#### Ruirong Yuan (yuan_rui@hotmail.com)

##### New Jersey Veteran’s Administration Medical Center, East Orange, NJ, USA

Therapy with immune checkpoint inhibitors has revolutionized cancer therapy. In the United States, four checkpoint inhibitors (nivolumab, pembrolizumab, atezolizumab and ipilimumab) have been approved for patients with previously treated melanoma, RCC, NSCLC and bladder cancer. Clinical data also shows that the programmed cell death 1 (PD-1) immune checkpoint inhibitor nivolumab has a better safety profile than anti-CTLA-4 ipilimumab. However, all currently approved checkpoint inhibitors when used as single-agent therapy only result in modest clinical improvement in patients with advanced disease. In this presentation, several topics will be discussed: 1. opportunities and challenges of immune checkpoint inhibitors for cancer treatment; 2. basic principles of cancer immune checkpoint inhibitor combination therapies; 3. future for cancer immunotherapies.

Many clinical trials evaluating different combination therapies are underway to determine if combinations can significantly improve overall response and long term survival in cancer patients.

**Fig. 2 (abstract A19).**
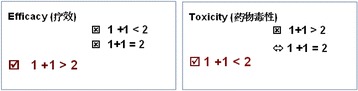
Principle of cancer immune checkpoint inhibitor combination therapies

### A20 Immunotherapy combination with radiotherapy

#### Bo Lu (bo.lu@jefferson.edu)

##### Thomas Jefferson University Sidney Kimmel Medical College, Philadelphia, PA, USA

PD-1inhibitors are approved to treat lung cancer and anti-PD1 therapy is poised to become a major front-line treatment for lung cancer. Integrating PD-1 inhibitors into radiotherapy regimens potentially breaks immune tolerance against lung cancer cells and synergistically activates T cells. However, it is not clear how these two modalities should be optimally combined. We have established novel mouse models that allow us determine the appropriate radiotherapy regimen and sequencing with anti-PD1 therapy. Similar questions are being addressed in an ongoing clinical trial. In addition, we investigated potential toxicities when thoracic radiotherapy is concurrently administered with anti-PD1 in a number of mouse models.

Activation of host immunity has the risk of enhancing radiation toxicities in normal tissues. Both cardiac and pulmonary toxicity were reported among patients receiving PD1 inhibitors. Both carditis and pneumonitis are evident in the PD-1null mice. Thoracic radiotherapy for patients with locally advanced lung cancer is associated with cardiac and lung toxicities stemming from an immune/inflammatory response. A number of reports including the analyses of the RTOG 0617 study suggested decreased survival in patients receiving more radiation exposure to the heart. Our preliminary data from mouse models demonstrated significantly increased acute death from thoracic or cardiac irradiation when PD-1 is absent or inhibited. We plan to translate our findings into better-designed clinical trials of anti-PD1 immunotherapy and radiotherapy for lung cancer.

## Session 7: Regulatory Considerations

### A21 Cancer immunotherapy: past, present and future

#### Ke Liu (keliunih@yahoo.com)

##### Chinese-American Hematologist and Oncologist Network (CAHON), New York, NY, USA

The last decade (2006–2016) has witnessed tremendous advances in cancer immunotherapy, illustrated by the increasing number of immunotherapeutic products approved by US FDA. Out of 184 oncological indications approved by US FDA, approximately one third indications were related to cancer immunotherapy [1]. The majority of approved indications are from monoclonal antibodies, the mainstay of the current hematologic and oncologic practices. The development of these antibodies has evolved considerably: from simple naked antibodies [2] to relatively complex conjugated antibodies [3], to bi-specific T cell engager (BiTE) [4], and to checkpoint inhibitor antibodies [5]. The latter two also redefine therapeutic monoclonal antibodies from merely “passive” immunotherapy to active immunotherapy since their mechanism of action involves activation of endogenous T cells. The approval for active immunotherapeutic agents has also undergone historical changes: from cytokines such IL-2 and interferon to more complicated cellular cancer vaccine [6] and oncolytic virotherapy [7]. Intensive efforts have been devoted to adoptive T cell therapy and chimeric antigen receptor (CAR) T cell therapy, neither of which has been approved in the US. Adoptive T cell therapy using tumor-infiltration lymphocyte (TIL) has been mainly studied in the advanced melanoma with response rates of 30-60 % [8]. CAR T cells have shown high response rates of 70-90 % in hematologic malignancies such as refractory or relapse acute lymphoblastic lymphoma (ALL) [9]. Challenges in the development of adoptive T cell therapy include chemistry, manufacturing and control and clinical considerations, such as study population, endpoint evaluation and toxicity management. The advent of modern technologies such as next gene sequencing has enabled cancer genome to be deciphered and let to the identification of neoantigen unique to the tumor [10]. When treated with immunotherapy, patients with tumors harboring more mutation load appear to have a better clinical outcome than patients with less mutation load [11]. Adoptive transfer of TIL containing mutation–reactive T cells could mediate the response of the tumor harboring the cognate antigen [12]. Active clinical research tests whether this approach as well as cancer vaccines based on the neoantigens have advantages over the shared antigens [13]. The approval of ipilimumab with nivolumab for advance melanoma ushers more venues for the power of immunotherapy [5]. More clinical research is being conducted for combination of immunotherapy.

**References**

1. http://www.fda.gov/Drugs/InformationOnDrugs/ApprovedDrugs/ucm279174.htm

2. Burstein HJ. N Engl J Med 2005;353:1652–1654

3. Verma S., et al., N Engl J Med 2012; 367:1783–1791

4. Hunger SP, et al., N Engl J Med 2015; 373:1541–1552

5. Lakin J., et al., N Engl J Med 2015; 373:23–34

6. Kantoff P., et al., N Engl J Med 2010; 363:411–422

7. Andtbacka R., et al., JCO 2015; 33: 2780–2788

8. Rosenberg SA, et al., Science 2015;348:62–68

9. Levine LB, Cancer Gene Therapy 2015 22: 79–84

10. Vogelstein B., et al. Science 2013;339:1546–1558

11. Rizvi NA. et al., Science 2015;348:124–128

12. Tran E., et al. Science 2014;344:641–645

13. Wu CJ., et al., Cancer Immunol Res July 2013 1; 11

### A22 Breakthrough therapy designation drug development and approval

#### Max Ning (ymcanning986@gmail.com)

##### Chinese-American Hematologist and Oncologist Network (CAHON), New York, NY, USA

Breakthrough Therapy designation is a regulatory program that was introduced in Section 902 of the Food and Drug Administration Safety and Innovation Act of 2012. Its objective is to expedite the development and review of investigational products intended to treat serious or life-threatening diseases, thereby improving patient early access to novel effective treatments. Designation of a Breakthrough Therapy is indication-based and requires preliminary clinical evidence that demonstrates the intended therapy or investigational product may provide substantial improvement over available or existing therapies on one or more clinically significant endpoints.

In oncology, 56 Breakthrough Therapies have been designated since the program’s inception^1^. Of them, 21 are already approved for clinical use. Six approved indications (28 %) are for immunotherapeutic products, suggesting that immunotherapy plays an important role in the Breakthrough Therapy program. For products approved between 2013 and 2015, median premarket development time was 2.2 years shorter among approved Breakthrough-designated products (12) than non-designated products (17)^2^. This preliminary analysis suggests that this program has considerably improved patient early access to novel effective cancer treatments.

Overall, the current evidence shows that Breakthrough Therapy designation has facilitated the development and review of novel effective oncology products, including immunotherapeutic products. Together with other expedited programs^3^, it helps address existing or emerging unmet medical needs in oncology. Most importantly, this program along with Accelerated Approval^4^ may substantially save time (e.g., 2–5 years earlier on average) for patients to access effective cancer treatments.

**References**

1. Breakthrough Therapies. Available at http://www.focr.org/breakthrough-therapies. Accessed June 2016.

2. Shea M, Ostermann L, Hohman R et al. Impact of breakthrough therapy designation on cancer drug development. Nature Review 2016; 15:152.

3. Guidance for Industry: Expedited Programs for Serious Conditions-Drugs and Biologics, available at: http://www.fda.gov/downloads/drugs/guidancecomplianceregulatoryinformation/guidances/ucm358301.pdf

4. Johnson JR, Ning YM, Farrell A, et al. Accelerated Approval of Oncology Products. JNCI 2011; 103:1–9.

### A23 Current European regulation of innovative oncology medicines: opportunities for immunotherapy

#### Harald Enzmann^1^, Heinz Zwierzina^2^

##### ^1^Federal Institute for Drugs and Medical Devices, Kurt-Georg-Kiesinger-Allee 3, 53175 Bonn, Germany; ^2^Cancer Drug Development Forum - CDDF Headquarters, c/o ECCO - Avenue E. Mounier 83, B-1200 Brussels, Belgium

###### **Correspondence:** Harald Enzmann (harald.enzmann@bfarm.de) – Federal Institute for Drugs and Medical Devices, Kurt-Georg-Kiesinger-Allee 3, 53175 Bonn, Germany

In the European Union (EU) regulatory agencies change their assessment procedures. The European Medicines Agency has instituted their Adaptive Pathways concept and priority medicines (PRIME) scheme for the early authorization of medicines (1). Early marketing authorization (MA) applications using Adaptive Pathways are based on surrogate endpoints confirmed by clinical outcome data post-authorization or on a selected subpopulation of most suitable patients for the initial application, expanding the indication later by variation procedures. PRIME will provide extensive regulatory support mostly in the presubmission phase and is expected to be most helpful for small and medium sized enterprises. Innovative oncology immunotherapies (IOI) are particularly well-positioned to profit from these changes and qualify for PRIME and adaptive pathways.

Regulators have become increasingly aware of the link between their assessment and pricing (3). Subsequent to the MA, mostly national decisions by Health Technology Assessment (HTA) bodies and payers have become prerequisites for patients’ access to innovative medicines. At variance with the Adaptive Pathways’ acceptance of surrogate endpoints HTA bodies put more emphasis on clinical outcome data (4). Within the EU, divergent decisions on reimbursement result in pronounced differences in patients’ access to innovative medicines. In addition, the time between MA and actual market launch is widely different between member states. The delay from MA to market launch is shortest for Germany the biggest market for medicines in the EU. The German system clearly separates three decision modules: 1. The European Medicines Agency’s assessment of the benefit risk balance and the MA decision. 2. The Federal Joint Committee’s assessment of the “additional benefit” of a medicine, i.e. its superiority to other therapeutic options available in Germany. 3. Price negotiations between marketing authorization holders and payers based on the medical need, the size of the additional benefit and the cost of the available alternative therapies, with potentially significant price corrections six or twelve months after marketing.

Joint scientific advice from regulators and HTA bodies will help to streamline development, optimize study design and support early MA and wide patients’ access. For innovative oncology immunotherapies particularly with potentially curative effect, PRIME and Adaptive Pathways may significantly facilitate and accelerate market access in the EU.

*Harald Enzmann and Heinz Zwierzina have no conflict of interest in relation to this article.*

**References**

1. European Medicines Agency, 2016a. PRIME: Priority Medicines. http://www.ema.europa.eu/ema/index.jsp?curl=pages/regulation/general/general_content_000660.jsp&mid=WC0b01ac05809f8439

2. Enzmann, H., New trends and challenges in the European regulation of innovative medicines, Regul Toxicol Pharmacol. 2016 May 27. pii: S0273-2300(16)30147-7. doi: 10.1016/j.yrtph.2016.05.033. [Epub ahead of print]

3. Eichler HG, Hurts H, Broich K, Rasi G: Drug Regulation and Pricing--Can Regulators Influence Affordability? N Engl J Med. 2016 May 12;374(19):1807–9.

4. Bergmann L, Enzmann H, Thirstrup S, Schweim JK, Widera I, Zwierzina H: Access to innovative oncology medicines in Europe. Ann Oncol. 2016 Feb;27(2):353–6.

